# Study on Erosion Wear Resistance of 18Ni300 Maraging Steel Remanufactured by Underwater Laser Direct Metal Deposition

**DOI:** 10.3390/ma18194583

**Published:** 2025-10-02

**Authors:** Zhandong Wang, Linzhong Wu, Shibin Wang, Chunke Wang

**Affiliations:** 1 College of Mechanical and Electronic Engineering, Nanjing Forestry University, Nanjing 210037, China; 3240300248@njfu.edu.cn (L.W.); ck_wang@njfu.edu.cn (C.W.); 2 School of Mechanical Engineering, Southeast University, Nanjing 211189, China; 230228059@seu.edu.cn; 3 Jiangsu Jiuxiang Automobile Electric Appliance Group Co., Ltd., Xuzhou 221225, China

**Keywords:** underwater direct metal deposition, additive manufacturing, maraging steel, microstructure, erosion wear, microhardness

## Abstract

Erosion wear is a major cause of surface degradation in metallic materials exposed to harsh marine environments. In this study, the erosion wear resistance of the 18Ni300 maraging steel repaired by underwater direct metal deposition (UDMD) is investigated. Results show that UDMD is successfully applied to repair the 18Ni300 samples in underwater environment. Full groove filling and sound metallurgical bonding without cracks are achieved, demonstrating its potential for underwater structural repair. Microstructural analyses reveal good forming quality with fine cellular structures and dense lath martensite in the deposited layer, attributed to rapid solidification under water cooling. Compared to in-air DMD, the UDMD sample exhibits higher surface microhardness due to increased dislocation density and microstructural refinement. Erosion wear behavior is evaluated at 30° and 90° impingement angles, showing that wear mechanisms shift from micro-cutting and plowing at 30° to indentation, crack propagation, and spallation at 90°. The UDMD samples demonstrate superior erosion wear resistance with lower mass loss, particularly at 30°, benefiting from surface work hardening and microstructural advantages. Progressive surface hardening occurs during erosion due to severe plastic deformation, reducing wear rates over time. The combination of refined microstructure, high dislocation density, and enhanced work hardening capability makes UDMD-repaired steel highly resistant to erosive degradation. These findings confirm that UDMD is a promising technique for repairing marine steel structures, offering enhanced durability and long-term performance in harsh offshore environments.

## 1. Introduction

Maraging steel is considered a critical material for deep-sea pressure resistance due to its extremely high strength, excellent toughness and ductility [1]. After aging heat treatment, fine and uniformly dispersed intermetallic compounds form within the martensitic matrix with high dislocation density. These compounds pin the movement of high-density dislocations in the martensitic matrix, resulting in second-phase strengthening [2,3]. During long-term service in harsh ocean environments, structural surfaces are susceptible to degradation caused by corrosion and wear, often resulting in surface pitting and crack formation. If left unaddressed, such damage can progress to catastrophic failure, severely compromising structural integrity and operational safety. Hence, effective repair strategies are critically needed.

Underwater arc welding is a widely used repair technique in marine engineering due to its operational flexibility and high repair efficiency [4,5,6]. However, it typically results in coarse microstructures and a large heat-affected zone, which can adversely affect the performance of the repaired components. In recent years, laser direct metal deposition (DMD), as a branch of additive manufacturing technology, has developed rapidly [7]. Characterized by high energy density, fine microstructure, minimal heat-affected zone, and excellent forming quality, DMD has found significant applications in the repair of high-value components in advanced equipment across various industrial sectors [8,9]. The DMD technique demonstrates significant potential for underwater repair applications.

Recently, numerous researchers have applied the DMD technique to underwater repair, aiming to restore the performance of the damaged components served in underwater environments. A novel technique is therefore proposed, namely underwater direct metal deposition (UDMD). Li et al. [10] developed a wire-based UDMD technique to repair S32101 duplex stainless steel in a nuclear power plant. They stated that the UDMD technique can meet the requirements of actual engineering for cladding layers. Fu et al. [11] investigated the mass transfer, microstructure evolution and mechanical properties of the Ti-6Al-4V repaired by UDMD. They found that the transformation pathway of the microstructure and resultant mechanical properties of the Ti-6Al-4V can be tailored by the heat input of UDMD. Cheng et al. [12] studied the influence of heat input on the microstructure and corrosion resistance of the high-strength steel fabricated by UDMD. They found that the corrosion resistance of underwater coatings is enhanced by the unique microstructure. Liao et al. [13] examined the microstructural features and mechanical properties of 308L stainless steel multi-layer components manufactured by UDMD. They demonstrated that the strong water-cooling effect leads to slender columnar crystals and higher ferrite contents, which impairs the strength and ductility of the UDMD 308L. Currently, research efforts are primarily focused on the correlation between the processing parameters, microstructure, and mechanical properties of UDMD components, while the service performance of materials in marine environments has received relatively less attention.

Maraging steel is subjected not only to high deep-sea pressure but also to complex service conditions such as fluid erosion wear. Erosive wear refers to the progressive loss of surface material from a solid subjected to relative motion with a fluid containing solid particles, driven by the synergistic action of mechanical abrasion and corrosion [14,15]. Wang et al. [16] investigated the erosion wear properties of 17-4PH stainless steel fabricated by laser additive manufacturing. They found that plastic deformation and micro-cutting are the main erosion mechanisms. Kumar et al. [17] studied the erosion resistance of the nitrogen-alloyed austenitic steel weld beads using a jet-type tester at different impingement angles. They found that the weight loss at a 30° impingement angle is non-uniform due to the low microhardness. In contrast, weight loss at a 90° impingement angle is uniform. Liu et al. [18] investigated the slurry erosion resistance of 0Cr12Ni9A and 0Cr17Ni4Cu4Nb coatings fabricated by high-power laser cladding. They found that the coatings with high hardness and strength exhibit excellent resistance to plastic deformation and failure when facing the impacts from sand particles. Zhao et al. [19] studied the slurry erosion resistance of the DMD martensitic age hardening steel treated at different aging temperatures. They found that the sample aged at 530 °C exhibits a significantly lower erosion rate, which is 6.25% of that in the as-built condition. Currently, studies on the erosion wear behavior of 18Ni300 steel are not available.

In our previous work [20], the microstructure and tensile strength of 18Ni300 maraging steel repaired using the UDMD technique were investigated. However, the erosion wear behavior of the repaired steel in actual marine environments has not been considered, which significantly hinders the practical application of underwater repair technology. In this study, the erosion wear behavior of UDMD-repaired 18Ni300 maraging steel is systematically examined. Erosion tests are conducted at impingement angles of 30° and 90° to evaluate the effect of angle on erosion wear performance. The evolution of mass loss rate and surface hardness as a function of erosion time is analyzed in detail. To further compare the influence of microstructural characteristics on erosion wear between in-air and underwater repaired samples, in-air repair of 18Ni300 steel is performed and its erosion wear performance is tested. This comparative analysis provides a comprehensive understanding of how microstructural features govern erosion wear resistance.

## 2. Experimental Procedure

### 2.1. Materials

**Figure 1:**
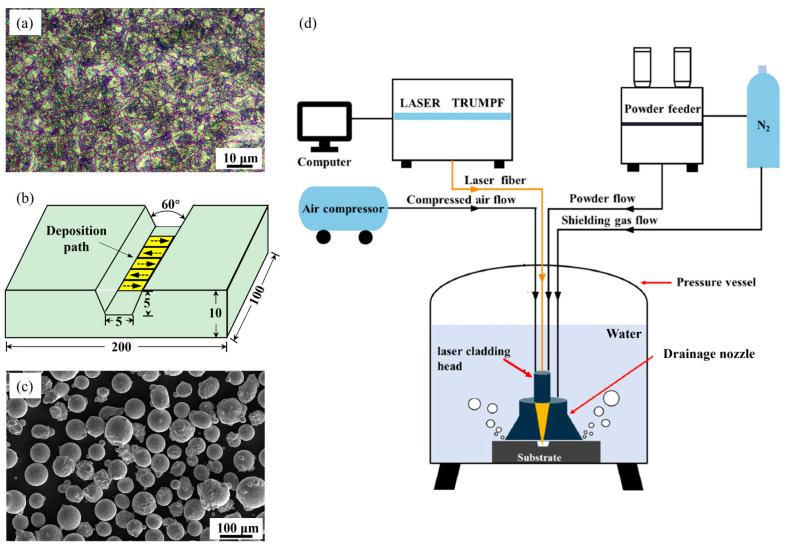
(**a**) Microstructure of the 18Ni300 substrate, (**b**) shape of the prepared trapezoidal groove, (**c**) SEM image showing the morphology of the 18Ni300 powder, and (**d**) schematic diagram showing the set-up of the UDMD system [20].

Rectangular 18Ni300 maraging steel substrates with dimensions of 200 × 100 × 10 mm are utilized, having undergone solution treatment followed by aging. The metallographic sample of the substrate is sequentially subjected to sandpaper grinding and standard mechanical polishing. Then it is etched with a 5% Nital solution (5 mL HNO_3_ + 95 mL ethanol). Figure 1a shows that the microstructure of the substrate is composed of aged martensite. The substrate features a lengthwise trapezoidal notch, as shown in Figure 1b. This notch is considered as the defects after manual mechanical shaping, which is convenient for underwater repair. Figure 1c shows the particle morphology of the 18Ni300 powder (from Avimetal Powder Metallurgy Technology Ltd., Beijing, China). Scanning electron microscope (SEM; Nova Nano, FEI Company, Hillsboro, USA) image shows that the powders have a spherical shape. Table 1 shows the chemical compositions (wt.%) of the 18Ni300 substrate and powder.

### 2.2. UDMD Set-Up

Figure 1d presents the experimental set-up of the UDMD system. The system mainly consists of a pressure vessel, a fiber laser device (Trudisk 6002, Trumpf, Ditzingen, Germany), a powder feeder (T003, Nanjing Huirui Photoelectric Technology Co., Ltd., Nanjing, China), and a three-axis motion platform [20]. The vessel is filled with water to simulate an underwater environment. A gas pressure of 0.3 MPa is applied, corresponding to a depth of 30 m. Repair is performed inside the vessel under this controlled pressure. A local dry zone is established on the surface of substrate using a home-made drainage nozzle. Laser cladding is carried out under this dry zone to prevent water interference. The 18Ni300 powder is fed coaxially through the cladding head and melted into the pool by the laser beam. The three-axis motion system controls the relative movement between the laser head and the substrate. It enables precise tracking of the programmed deposition path, as shown in Figure 1b.

### 2.3. UDMD Process Parameters

The optimal process parameters for UDMD repair are determined through preliminary experiments. The power of the laser is set to 3500 W. The velocity of the laser cladding head is set to 1167 mm/min. The feed rate of the powder particles is set to 26 g/min. The diameter of the laser beam is 3 mm. The overlap rate of the adjacent tracks is 50%. A total of 4 layers are deposited to fill the groove with a height of 5 mm. The height moved by each layer is set to 1.25 mm. DMD is also conducted in ambient air. The same parameters are applied in this case. This condition is referred to as in-air DMD. It serves as a reference to evaluate the effect of environmental conditions on the repair performance.

### 2.4. Microstructure Characterization

**Figure 2:**
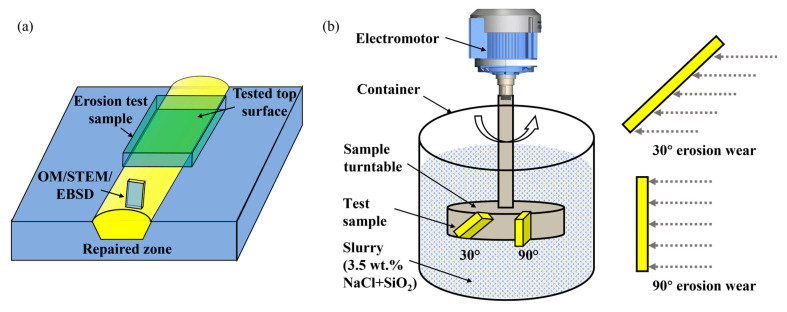
(**a**) Sampling positions for the microstructural characterization, and (**b**) schematic diagram showing the erosion wear test.

The repaired zone (RZ) is cut from the steel plate by electrical discharge machine (EDM; HB400X, Suzhou Sanguang Science &Technology Co., Ltd., Suzhou, China), as shown in Figure 2a. After grinding and polishing, a 5% Nital solution is used to etch the RZ. Microstructure of the RZ is characterized by optical microscopy (OM; Primotech, Carl Zeiss, Oberkochen, Germany), scanning-transmission electron microscopy (STEM; Talos F200X, Thermo Fisher Scientific, Waltham, USA) and electron backscatter diffraction (EBSD; Oxford Instruments, Abingdon, UK). The step size for EBSD is 0.3 μm.

### 2.5. Erosion Wear Test

The erosion wear experiment is conducted under the guidance of the YB/T 6177-2024 standard [21]. The specimen used for the erosion wear test had a dimension of 20 mm × 30 mm × 8 mm. The specimen was extracted from the repaired steel plate using EDM. The cutting position is shown in Figure 2a. The tested surface is ground with sandpapers and then mechanically polished. Then, the specimen is ultrasonically cleaned with absolute ethyl alcohol and dried. The erosion wear test is conducted using a solid–liquid dual-phase erosion wear system (MCF-20, Jinan Outuo Experimental Equipment Co., Ltd., Jinan, China). The system consists of an electromotor, a container, a sample turntable and slurry, as shown in Figure 2b. The jet impinges on the sample surface at different angles by rotating the sample turntable. The parameters for the erosion wear test are summarized in Table 2.

Erosion wear tests are conducted on DMD and UDMD repaired specimens at erosion angles of 30° and 90° to investigate the erosion wear mechanism. For convenience, the samples are labeled as UDMD-30°, UDMD-90°, in-air DMD-30°, and in-air DMD-90°, respectively. This work utilizes the volumetric wear rate (E) to characterize the wear volume per unit time per unit area of the material, which can be calculated using Equation (1):

(1) $$E=\frac{\Delta m}{S\cdot t\cdot \rho }$$

where Δm is the wear mass, S is the erosion area, t is the erosion time, and ρ is the sample density. The mass of all test specimens is measured using an electronic balance with an accuracy of 0.0001 g. After every hour of erosion, the specimens are weighed to quantitatively analyze the intrinsic relationship between mass loss and erosion time. To minimize experimental error, all specimens are ultrasonically cleaned with absolute ethyl alcohol and dried. To further observe the erosion process and analyze the erosion mechanisms, the microscopic morphology of the eroded areas is characterized using SEM every 2 h of erosion.

### 2.6. Surface Contour Characterization

**Figure 3:**
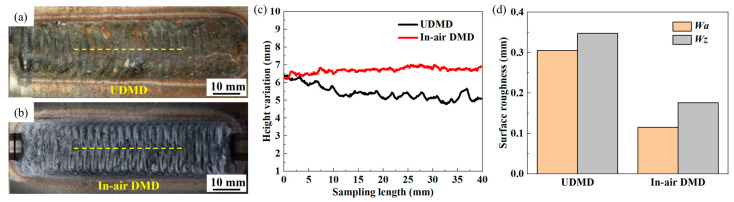
(**a**,**b**) Top surface morphology of the UDMD and in-air DMD repaired samples, (**c**) surface contour evolution of the yellow dashed lines marked in (**a**), and (**d**) average surface roughness (Sa and Sz) values of the samples.

A profilometer (SJ5718, Zhongtu Instruments Co., Ltd., Shenzhen, China) is employed to measure the macro surface profile of the RZ. The sampling length for the macro surface profile measurement is set to 40 mm, with a movement speed of 5 mm/s. The sampling position is selected at the center of the RZ, as shown in Figure 3a,b. Two distinct parameters are employed to evaluate the fluctuation degree of the macro surface profiles: the maximum height of the profile (*Wz*) and the arithmetic mean deviation of the profile (*Wa*). In addition, after 6 h erosion wear test, the micro surface morphology of the samples is characterized using a three-dimensional confocal laser scanning microscope (CLSM; LSM900, Carl Zeiss, Oberkochen, Germany).

### 2.7. Microhardness Test

The microhardness distribution on the surface of RZ before and after erosion wear test is measured by a Vickers indenter (HXD-1000 TMSC/LCD, Shanghai Taiming Optical Instrument Co., Ltd., Shanghai, China). The indentation is performed under a 200 gf load with a 10 s holding period. The microhardness is averaged by the values of ten measured points.

## 3. Results and Discussion

### 3.1. Surface Morphology

Figure 3a,b show the top surface morphology of the UDMD and in-air DMD 18Ni300 samples. The trapezoidal grooves in both samples are completely filled with the deposited layers, exhibiting good repair quality without obvious surface defects such as cracks or lack of fusion. This demonstrates the feasibility of on-site repair using additive manufacturing technology in underwater environments. Figure 3a,b show that the both RZ display a continuous fish-scale-like pattern, which is caused by the Z-shaped deposition strategy. Compared with the in-air DMD sample, the surface of the UDMD sample is covered by rust. When the high-temperature deposits are immersed in the water, intense oxidation occurs on the top surface. The oxidized steel surface is quickly corroded in the water environment [22].

Figure 3c shows measurement results of the surface profiles of the yellow dashed lines marked in Figure 3a,b. The surface profile fluctuation of the UDMD sample is greater than that of the in-air DMD sample. As shown in Figure 3d, the *Wa* and *Wz* values of the UDMD sample are higher than those of the in-air DMD sample. This phenomenon can be explained by the following factors. On the one hand, the heat dissipation in the underwater environments is fast. There is less thermal accumulation in the molten pool, leading to a rapid solidification rate. As a result, the liquid molten pool quickly solidifies before it has sufficient time to flow and wet the substrate, resulting in a poor surface quality. On the other hand, the presence of the high-flow-rate drainage gas and nozzle gases changes the flow field above the underwater molten pool. This generates more spatters with high velocity. In addition, a great number of partially melted powder particles are adhered on the surface of the deposited layer. These factors cause the great surface fluctuations of the UDMD sample.

### 3.2. Microstructure of Repaired Zone

**Figure 4:**
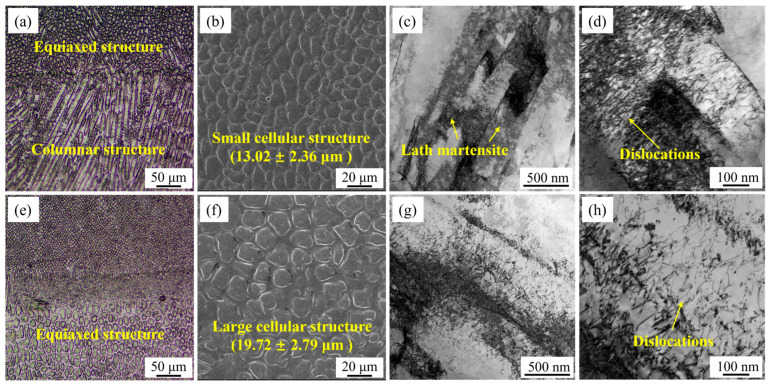
Multi-scale microstructural features of the (**a**–**d**) UDMD sample and (**e**–**h**) in-air DMD sample. (**a**,**e**) OM images, (**b**,**f**) SEM images, and (**c**,**d**,**g**,**h**) TEM images.

Figure 4 shows the multi-scale microstructural features of the UDMD and in-air DMD samples. Figure 4a shows that the solidified microstructure of the UDMD RZ exhibits a cellular morphology with different columnar and equiaxed shapes. Different microstructural morphologies are due to the fact that the growth direction of microstructure is disordered in the three-dimensional molten pool. Similar observations have been reported in previous studies [23]. The formation of the cellular structure phenomenon is caused by the rapid solidification rate of the molten pool, which leads to solidification before secondary dendrite arms can develop. Figure 4e shows that the solidified microstructure of the in-air DMD RZ has an equiaxed shape. Figure 4b,f show that the size of the cellular structure in the UDMD sample is smaller than that in the in-air DMD sample. This is owing to the relatively fast cooling rates involved in the UDMD process. The presence of water cooling and forced gas cooling contributes to the fast solidification of the molten pool. TEM results show that the microstructure of the RZ is composed of lath martensite, as shown in Figure 4c. The size of the lath martensite in the UDMD sample is smaller than that in the in-air DMD sample (Figure 4g). Figure 4d,h show that the dislocation density in the UDMD sample is higher than that in the in-air DMD sample. The fast cooling rate in the UDMD sample causes rapid contraction of the solidified metal, inducing significant plastic strain. This results in a high density of dislocations to accommodate the strain [24].

In contrast to the water-cooled UDMD process, the in-air DMD process provides less effective heat dissipation. This leads to increased peak temperatures and a significantly slower cooling profile. This thermal environment promotes prolonged austenitization and allows for greater grain growth, resulting in a coarser microstructure. Furthermore, the extended exposure to elevated temperatures facilitates more extensive recovery and tempering processes, leading to a reduction in dislocation density within the in-air DMD sample.

**Figure 5:**
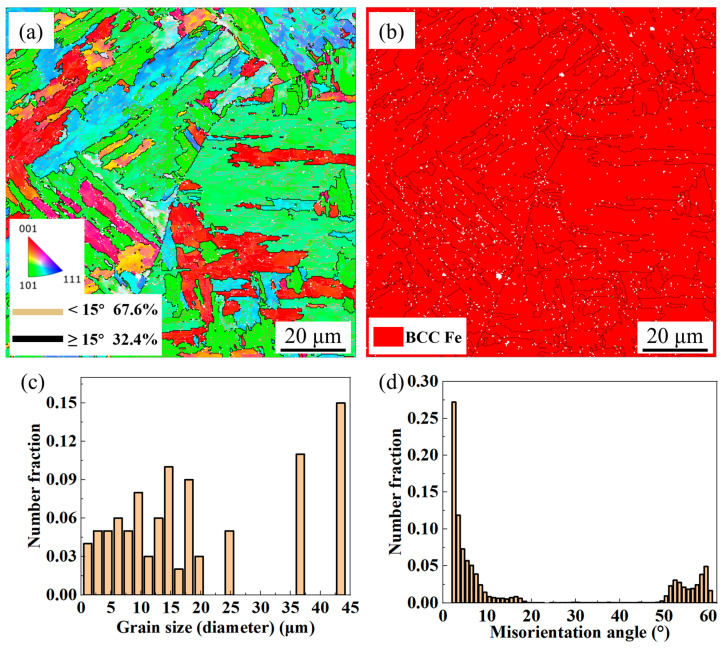
EBSD results of the UDMD 18Ni300 microstructure. (**a**) Inverse pole figure (IPF) map, (**b**) phase map, (**c**) grain size distribution, and (**d**) misorientation angle distribution.

Figure 5 shows the EBSD measurement results of the UDMD 18Ni300 microstructure. The IPF map reveals that the cellular structure exhibits various orientations, indicating the absence of a strong texture in the solidified 18Ni300, as shown in Figure 5a. Similar observation is reported in Ref. [25]. The fractions of high-angle grain boundaries (HAGBs, >15°) and low-angle grain boundaries (LAGBs, 2–15°), represented by green lines, are 32.4% and 67.6%, respectively. The grain interiors are rich in substructures, including dislocations and lath martensite boundaries. Figure 5b shows that the microstructure consists of the BCC phase, with no residual austenite detected in the inter-dendritic regions [26,27]. Figure 5c indicates that the main grain sizes are less than 25 μm. Figure 5d shows that the misorientation angles are predominantly concentrated at low-angle grain boundaries, with the highest fraction at 2° (27%), corresponding to the high density of lath boundaries.

### 3.3. Erosion Wear Performance

**Figure 6:**
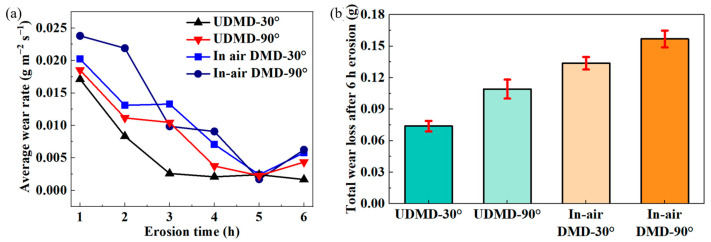
(**a**) Average wear rate with the erosion time and (**b**) total wear mass loss after 6 h erosion testing.

The volumetric wear rate per hour for each sample is calculated according to Equation (1) to evaluate the erosion wear resistance. The results are shown in Figure 6a. Under the same erosion parameters, the volumetric wear rates of the DMD samples are relatively higher than those of the UDMD samples. Meanwhile, the volumetric wear rates at the erosion angle of 90° are higher than those at the erosion angle of 30°, indicating a maximum wear rate at 90°. This confirms that the repaired 18Ni300 samples exhibit a typical brittle erosion wear characteristic. Moreover, the volumetric wear rate of all samples tends to decrease with increasing erosion time. This is due to the surface strengthening from the severe plastic deformation caused by the continuous surface impact. The longer the erosion time, the stronger the surface microstructure.

Figure 6b shows the total wear mass loss of each sample after 6 h of erosion testing. The highest total mass loss occurs in the in-air DMD sample eroded at 90°, while the lowest mass loss is observed in the UDMD sample eroded at 30°. The total mass loss of the eroded samples in ascending order is UDMD-30° < UDMD-90° < in-air DMD-30° < in-air DMD-90°, with values of 0.0739 g, 0.1091 g, 0.1337 g, and 0.1568 g, respectively. The total wear mass of the UDMD sample is lower than that of the in-air DMD sample, demonstrating that UDMD 18Ni300 possesses superior erosion wear resistance.

**Figure 7:**
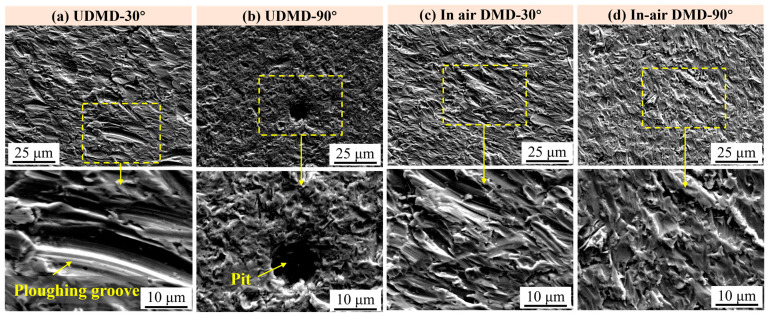
Surface morphology after 2 h erosion wear testing. (**a**) UDMD-30°, (**b**) UDMD-90°, (**c**) in-air DMD-30°, and (**d**) in-air DMD-90°.

The surface morphologies of eroded samples after different erosion times are examined using SEM to further analyze the erosion wear process and the underlying wear mechanisms. Figure 7 shows the typical surface morphologies of the eroded samples after 2 h testing. Following 2 h of exposure to quartz sand particles, all samples exhibit varying degrees of erosion damage. Figure 7a presents that the surface is featured by numerous grooves, primarily caused by cutting and plowing actions as the sand particles impact the surface at an acute angle. Some surface material is directly removed by cutting, while other portions are displaced and piled up on both sides of the grooves. In contrast, Figure 7b shows that the UDMD-90° exhibits craters and pits on its surface due to the dominant normal impact force. Under repeated high-stress impacts, the surface material undergoes reduced plasticity, leading to microcrack initiation at stress concentration sites. As these cracks propagate, spalling of material occurs, resulting in large-scale delamination. Moreover, the circular shaped pitting feature is related to the joint effect of erosion and corrosion, which is a common erosion-corrosion defect on the surface of steels [28]. The typical erosion morphologies of in-air DMD samples are similar to those of UDMD samples. However, the erosion patterns on in-air DMD samples appear more uniform compared to those on UDMD samples.

**Figure 8:**
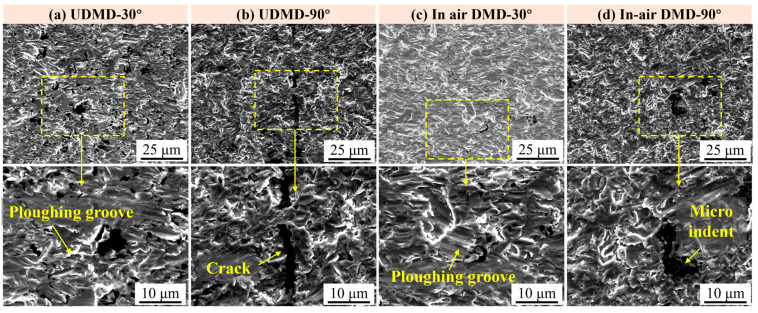
Surface morphology after 4 h erosion wear testing. (**a**) UDMD-30°, (**b**) UDMD-90°, (**c**) in-air DMD-30°, and (**d**) in-air DMD-90°.

Figure 8 shows the surface morphologies of the samples after 4 h erosion testing. In addition to the typical erosion characteristics caused by quartz sand impacts, white corrosion products can also be observed due to corrosion effects. For samples eroded at a 30° angle (Figure 8a,c), besides the grooves formed by micro cutting and plowing actions of the erosive particles, there is also evidence of surface fragmentation and material spalling due to the relatively low toughness of the surface. In addition, for samples eroded at a 90° angle (Figure 8b,d), the erosion wear appears more severe, with deeper craters resulting from the normal impact forces. As the erosion time increases from 2 h to 4 h, repeated impacts lead to a continuous reduction in ductility and accumulation of residual stresses, causing cracks to initiate at defect sites and leading to further surface spalling.

**Figure 9:**
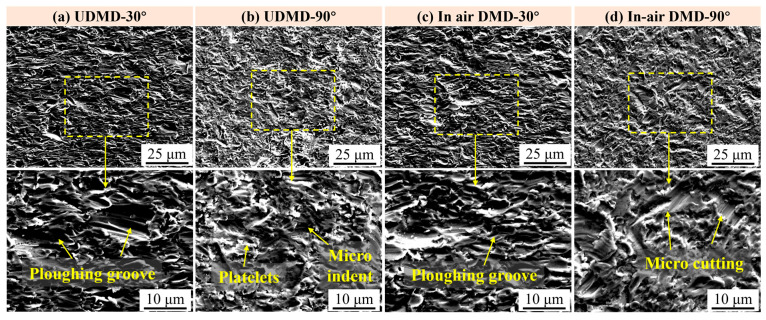
Surface morphology after 6 h erosion wear testing. (**a**) UDMD-30°, (**b**) UDMD-90°, (**c**) in-air DMD-30°, and (**d**) in-air DMD-90°.

Figure 9 shows the surface morphologies of the samples after 6 h erosion testing. Compared to the surface morphologies after 2 h and 4 h erosions, the main wear morphology of the repaired samples after 6 h erosion do not change significantly, but the degree of wear becomes more severe. Specifically, Figure 9a,c show the surface morphologies of UDMD-30° sample and in-air DMD-30° sample, respectively. By comparing these with the surface morphologies after 2 h (Figure 7a,c) and 4 h (Figure 8a,c) erosion, it is observed that due to the cutting and plowing actions of erosive particles, some particles deepen existing grooves, while others create new grooves at the protruding edges adjacent to the grooves. This results in an increase in both the number and depth of grooves as the erosion time extends. Figure 9b,d show the surface morphologies of UDMD-90° sample and in-air DMD-90° sample, respectively. With increasing erosion time, craters and indents formed by normal impact forces become deeper and surface fragmentation becomes more severe. In addition, platelets are formed due to continuous extrusion-forging on the plastically deformed surface [29].

**Figure 10:**
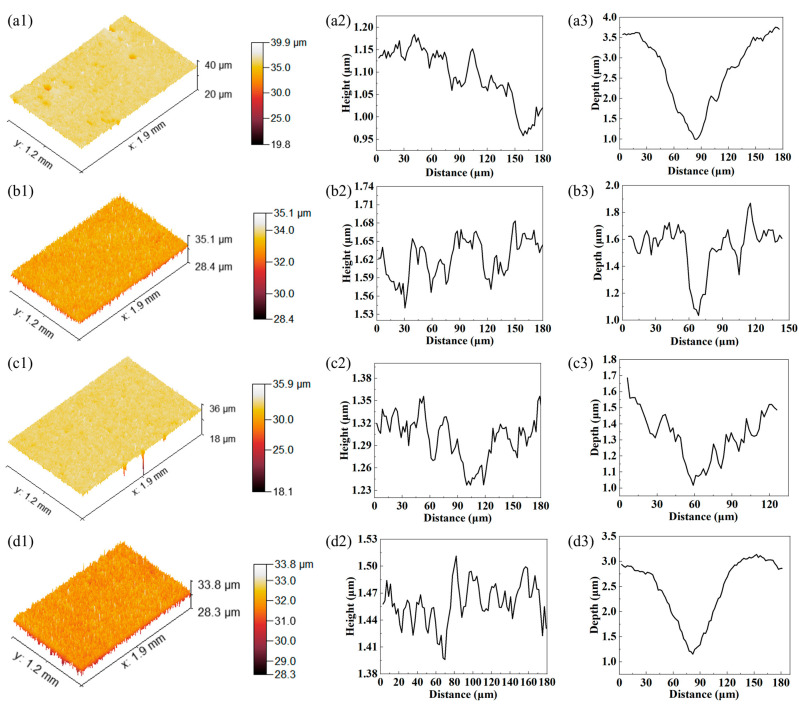
CLSM images showing the surface morphology of the sample after 6 h erosion wear testing. (**a**) UDMD-30°, (**b**) UDMD-90°, (**c**) in-air DMD-30°, and (**d**) in-air DMD-90°. (**a1**–**d1**) Three-dimensional profiles of the eroded surface. (**a2**–**d2**) Profile fluctuation of the eroded surface. (**a3**–**d3**) Profile of a typical micro indent on the eroded surface.

Figure 10 shows the three-dimensional topography of samples after 6 h of erosion wear testing. Figure 10(a1–d1) show that the height fluctuation on the 30° eroded surface is greater than that on the 90° eroded surface. This is mainly due to the fact that the cutting effect of oblique angle erosion generates plowing grooves with large height differences. For the 90° erosion surface, the impact process by the quartz sand particles is more uniform. The removal rate of materials at the surface is also more uniform, leading to a smaller height difference. Figure 10(a2–d2) shows the surface profiles without micro indents, which reflects the surface mechanical damage by the quartz sand particles. Figure 10(a2,b2) show that the maximum profile fluctuations of the UDMD-30° and UDMD-90° samples are 0.23 μm and 0.13 μm, respectively. Figure 10(b2) shows that the surface fluctuation in the UDMD-90° sample is uniform, which agrees well with the 3D measurement result (Figure 10(b1)). Figure 10(c2,d2) show that the maximum profile fluctuations of the in-air DMD-30° and in-air DMD-90° samples are 0.12 μm and 0.11 μm, respectively. Compared with the UDMD sample, the profile fluctuation in the in-air DMD sample is small. Figure 10(a3–d3) show the depth and width of typical micro indents on the erosion surface. The width of the pits ranges from 45 μm to 120 μm, while the depth of the micro indents ranges from 0.6 μm to 2.5 um. The experimental measurements indicate that the size distribution of the micro indents formed by erosion wear is random.

### 3.4. Erosion Wear Mechanism

**Figure 11:**
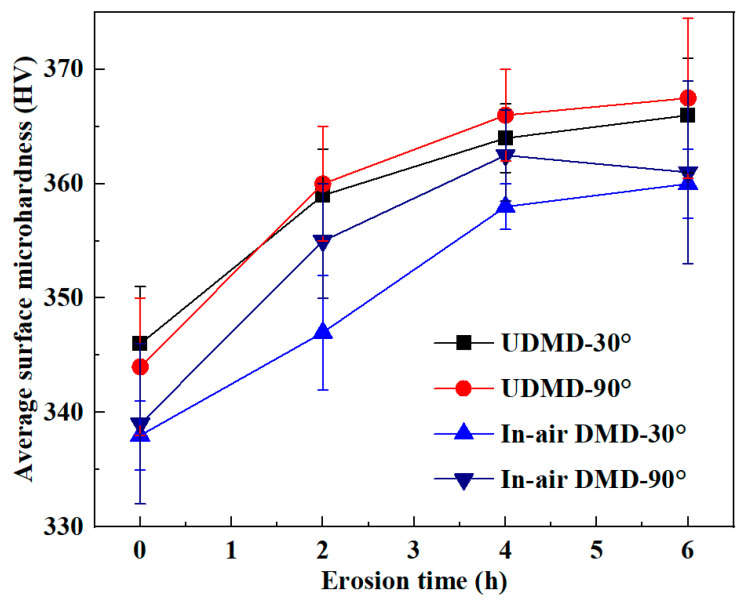
Surface microhardness changes with erosion time.

By comparing the erosion wear volumes and analyzing the erosion wear morphologies of the samples, it can be seen that the dominant erosion wear mechanisms of DMD-repaired and UDMD-repaired samples are similar. Generally, the resistance to erosion wear is closely related to microhardness and toughness of the material. Figure 11 shows the microhardness changes with the increase in erosion time. The results show that the microhardness of all eroded samples increases with increasing erosion time. Throughout the erosion process, the UDMD samples consistently exhibit marginally higher microhardness compared to their in-air DMD counterparts. These findings can explain two key observations. One is the decreasing trend in volumetric wear rate over time. The other is the lower total mass loss exhibited by the UDMD samples compared to the in-air DMD samples.

During the erosion wear process, repeated and sustained impacts from quartz sand particles induce severe plastic deformation on the material surface, leading to grain slip, dislocation tangling, and elongation, as well as fragmentation and fibering of grains [30]. These microstructural changes, along with the accumulation of residual stresses within the topmost surface region, result in a work hardening effect. This effect is characterized by an increase in strength and microhardness, accompanied by a reduction in ductility and toughness [31,32].

**Figure 12:**
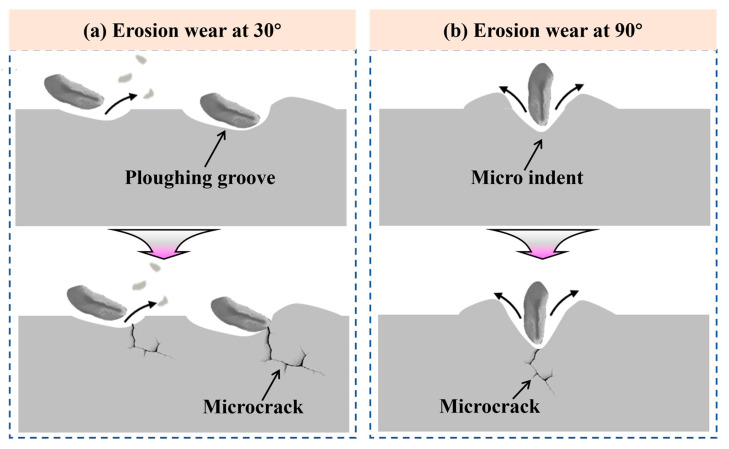
Schematic diagrams showing surface evolution mechanisms of the repaired 18Ni300 subjected to erosion wear testing: (**a**) 30° and (**b**) 90°.

Figure 12 shows the erosion wear mechanisms of the repaired 18Ni300 samples. During the erosion wear process, a large number of quartz sand particles continuously impact the sample surface. The impact force can be resolved into a tangential component (parallel to the surface) and a normal component (perpendicular to the surface). The tangential force causes grooves on the material surface through plowing and cutting, resulting in partial material loss. The normal force induces indentation deformation, which promotes crack initiation and subsequent material spallation [33].

At an erosion angle of 30°, the tangential force dominates, as shown in Figure 12a. In the initial stage of erosion, the kinetic energy of the quartz sand particles is primarily converted into strain energy, leading to plastic deformation and dislocation multiplication in the eroded sample. Some particles penetrate the surface and displace material to the sides and ends of their trajectories, forming plowed ridges and grooves. Other particles directly remove surface material through a cutting action [34]. As erosion progresses, when the accumulated plastic deformation exceeds the plastic deformation capacity, microcracks initiate at defects or stress concentration sites (Figure 12a). The continued tangential cutting action accelerates the propagation of these microcracks, leading to flake-like or chunk-like material detachment and the formation of spallation pits.

At an erosion angle of 90°, the sample surface is subjected primarily to the normal component of the impact force, as shown in Figure 12b. This results in more severe plastic deformation. In the initial stage of erosion, the normal impact of quartz sand particles creates craters on the surface, displacing material to the periphery of the indentations. As the sand particles continue to impact the surface, these craters grow larger and deeper, leading to stress concentration and promoting the initiation of microcracks (Figure 12b). Repeated impacts cause the microcracks to propagate and coalesce into larger cracks, ultimately resulting in surface fracture and material spallation.

## 4. Conclusions

In this study, the erosion wear performance of 18Ni300 maraging steel fabricated by underwater direct metal deposition (UDMD) is investigated. The relationships among processing conditions, microstructural characteristics, microhardness, and erosion wear performance are systematically established. Furthermore, the erosion wear mechanisms at different impingement angles are elucidated. The main conclusions are summarized as follows.

(1) UDMD can effectively restore damaged components with full groove filling and sound metallurgical bonding, confirming its technical feasibility for underwater structural repair in marine engineering applications.
(2) Microstructural analysis revealed that the UDMD process, under rapid water cooling and gas shielding, produces finer cellular structures and denser lath martensite with higher dislocation density compared to in-air DMD. This refined microstructure is attributed to accelerated solidification rates, which enhance the microhardness and contribute to improved surface integrity.
(3) Erosion wear tests at 30° and 90° impingement angles reveal distinct wear mechanisms: oblique impacts cause cutting and plowing, while normal impacts induce indentation, cracking, and spallation. The UDMD samples exhibit lower volumetric wear rates and total mass loss, particularly at 30°, indicating superior erosion resistance due to their finer microstructure and higher hardness.
(4) Surface hardening occurs progressively during erosion due to severe plastic deformation and dislocation accumulation, leading to reduced wear rates over time. The combination of microstructural refinement, high dislocation density, and work hardening in UDMD-repaired 18Ni300 steel provides excellent resistance to erosive wear in harsh marine environments, making it a promising solution for long-term underwater structural integrity.

## Figures and Tables

**Table 1:** Chemical compositions (wt.%) of the 18Ni300 substrate and powder.

**Element**	**C**	**Ni**	**Co**	**Mo**	**Al**	**Ti**	**Si**	**Fe**
18Ni300 substrate	0.023	18.1	8.6	5.5	0.13	0.49	0.04	Bal.
18Ni300 powder	0.005	17.9	9.1	5.0	0.11	0.80	0.03	Bal.

**Table 2:** Process parameters for erosion wear test.

**Parameter**	**Value**
Erosion angle	30° and 90°
Erosion particles	5 wt.% quartz sand
Particle size	125–700 μm
Erosion solution	3.5 wt.% NaCl solution
Erosion time	2 h, 4 h and 6 h
Erosion velocity	10 m/s

## Data Availability

The original contributions presented in this study are included in the article. Further inquiries can be directed to the corresponding authors.

## Abbreviations

The following abbreviations are used in this manuscript:

UDMD	Underwater direct metal deposition
In-air DMD	In-air direct metal deposition
RZ	Repaired zone
EDM	Electrical discharge machine
SEM	Scanning electron microscope
OM	Optical microscope
STEM	Scanning-transmission electron microscope
EBSD	Electron backscatter diffraction
CLSM	Confocal laser scanning microscope
HAGBs	High-angle grain boundaries
LAGBs	Low-angle grain boundaries
